# E-Mental-Health aftercare for children and adolescents after partial or full inpatient psychiatric hospitalization: study protocol of the randomized controlled DigiPuR trial

**DOI:** 10.1186/s13063-022-06508-1

**Published:** 2022-08-26

**Authors:** Marlene Finkbeiner, Jan Kühnhausen, Johanna Schmid, Annette Conzelmann, Ute Dürrwächter, Lena-Marie Wahl, Augustin Kelava, Caterina Gawrilow, Tobias J. Renner

**Affiliations:** 1 Department of Child and Adolescent Psychiatry, Psychosomatics and Psychotherapy, University Hospital of Psychiatry and Psychotherapy, Osianderstrasse 14-16, 72076 Tuebingen, Germany; 2 PFH – Private University of Applied Sciences, Department of Psychology (Clinical Psychology II), Weender Landstraße 3-7, 37073 Goettingen, Germany; 3 Methods Center, University of Tuebingen, Hausserstrasse 11, 72076 Tuebingen, Germany; 4 Department of Psychology, University of Tuebingen, Schleichstrasse 4, 72076 Tuebingen, Germany

**Keywords:** Aftercare, Child and adolescent psychiatric hospitalization, E-Mental Health, Preventing rehospitalization, Randomized controlled trial, School reintegration

## Abstract

**Background:**

During reintegration to daily school life following psychiatric hospitalization, children and adolescents are confronted with various challenges and are at risk for rehospitalization. Tailored post-discharge services could support a successful readjustment and accompany the high-risk transition period after discharge. The study DigiPuR (“Digital gestützte Psychotherapie und Reintegration,” digitally supported psychotherapy and reintegration) aims to establish and to evaluate an innovative digital aftercare program to alleviate challenges during reintegration and improve cross-sectoral care.

**Methods:**

DigiPuR is a randomized controlled trial comparing a digital aftercare service with regular aftercare (TAU) (planned *N* = 150, 25 children/adolescents, 25 parents, and 25 teachers in each group). In the intervention group, direct communication via secure and regular video calls until 8 weeks after discharge and a secure messenger system between the hospital, family, and school, as well as, if needed, external support systems, are established. A longitudinal pre-post-follow-up assessment at admission, discharge, and 8, 24, and 36 weeks after discharge takes place supplemented by a daily smartphone-based ambulatory assessment from a triadic perspective of patients, parents, and teachers. Primary outcomes include whether participants in the intervention group have fewer readmissions and higher treatment satisfaction and health-related quality of life as well as lower symptom severity than participants in the control group.

**Discussion:**

The present study is essential to address the cross-sectoral challenges associated with reintegration into daily (school) life following child and adolescent psychiatric hospitalization and to determine possible needed adaptations in partial or full inpatient settings. If applicability and efficacy of the aftercare service can be demonstrated, integration into regular care will be sought.

**Trial registration:**

ClinicalTrials.govNCT04986228. Registered on August 2, 2021

**Supplementary Information:**

The online version contains supplementary material available at 10.1186/s13063-022-06508-1.

## Background

The prevalence of mental disorders among children and adolescents in Germany and worldwide is between 13 and 18% [1, 2]. For the treatment of mental disorders, in addition to outpatient services, there are partial and full inpatient services as more intensive types of treatment [3]. In 2020, 53,436 patients received full inpatient treatment in child and adolescent psychiatry in Germany [4]. Correspondingly, utilization of the limited inpatient beds in the area of child and adolescent psychiatry is high, with about 89% of available beds in Germany in 2019 being in use [5]. Besides the two areas of general psychiatry and psychosomatics/psychotherapy, this figure is among the highest utilization rates in all departments in German hospitals [5]. Rehospitalization rates have been reported to range from 12 to 57% with percentages tending to be higher for longer follow-up periods [6–12], but most readmissions occurring within the first 3 months after discharge [8–10].

After discharge following psychiatric hospitalization, children and adolescents are confronted with various challenges, emerging predominantly in the academic, social, and emotional domain [13–16]. Academically, children and adolescents need to make up missed school work, with some additionally transferring to a new class or school [14–16]. Socially, children and adolescents report fears of explaining their absence, concerns to lose friendships, and experiences of bullying and stigmatization [14–17]. Emotionally, despite substantial symptom reduction during inpatient treatment, intense negative or overwhelming emotions and residual symptoms often persist or reappear after discharge [14–16, 18, 19]. About 87% of children and adolescents require further assistance after discharge [20]. A seamless transition to outpatient follow-up treatment is desirable, as transfer of therapeutically achieved treatment progress into daily situations usually succeeds only with targeted practice and accompaniment, due to the context dependence of symptomatic behavior [21, 22]. In practice, however, long waiting times, in Germany averaging 5 weeks for an initial consultation and 18 weeks to begin outpatient psychotherapy [23], often prevent a seamless transition between treatment sectors. In case of a child or adolescent starting treatment with a previously unknown outpatient psychotherapist, a sustainable therapeutic relationship, as well as motivation for further treatment, must be newly established, further complicating the transition between inpatient and outpatient care.

Important attachment figures, such as parents or teachers, can help children and adolescents cope with transition-related challenges [24], and it is important to involve them in inpatient psychiatric treatment [25]. Higher parental involvement is associated with a lower risk of the child being rehospitalized [9]. Furthermore, worsening student-teacher relationships as well as increased bullying experiences after hospitalization are associated with higher levels of suicidal ideation in the immediate post-discharge period [26]. However, for many attachment figures, a child’s psychiatric hospitalization is also associated with strain and distress and they often report feelings of incompetence and uncertainties about how to suitably support the child [13, 19, 27, 28]. Supporting attachment figures in the form of providing psychoeducation, strengthening resources, and developing competencies and coping strategies so that they can better understand the child’s situation and adequately deal with it is required and helpful for a successful reintegration [13, 24, 27].

Sufficient and tailored post-discharge support services for patients and their attachment figures could support a successful readjustment and accompany the challenging transition period after discharge [13, 29]. Ideally, case-leading therapists of inpatient treatment seamlessly accompany the reintegration phase for some more weeks after discharge and support readjustment to daily (school) life as well as transfer of treatment successes. The case-leading therapists know the case and all parties involved from the former treatment and can therefore offer support to all parties within the existing therapeutic relationship. This, however, requires sufficient monetary and time resources from all stakeholders.

E-Mental-Health services support people with mental strains through information and communication technologies [30] and offer enormous potential to improve health care [31, 32]. Advantages include a wide reach for treatment services, lack of travel time with more flexible and tailored scheduling for appointments, and low-threshold services [33, 34]. Moreover, the created distance, for example in therapy via videoconferencing, can lead to less dependence on the therapist and to greater disinhibition in statements as well as to additional information by seeing patients in their home environment, which can be helpful for treatment [33]. Demand and use for E-Mental-Health services has once again considerably increased and taken on particular relevance since the beginning of the COVID-19 pandemic [33, 35, 36]. Especially in the context of aftercare, E-Mental-Health services can complement inpatient treatment and close the gap to outpatient treatment by relying on existing therapeutic relationships to seamlessly support the transition from psychiatric hospital to daily life in a low-threshold manner [34, 37]. A recent review on discharge interventions from inpatient child and adolescent mental health care identified only two randomized controlled trials in this area, none of which examined a digitally supported intervention [29]. However, one feasibility study discussed in the review [29] showed a general interest in downloading a smartphone-based application for a digital safety plan at the time of discharge [38]. Studies on the efficacy of digital aftercare services for adults suggest beneficial impacts on patients’ post-discharge development [39–43].

The innovative aftercare program DigiPuR (“Digital gestützte Psychotherapie und Reintegration”, digitally supported psychotherapy and reintegration) aims to stabilize and ideally expand treatment successes after partial or full inpatient child and adolescent psychiatric treatment, therefore reducing a worsening of symptoms and rehospitalizations. In the intervention group, direct communication via secure and regular video calls and a messenger system between the hospital, family, and school, as well as, if needed, external support systems, are established. Thereby, problems can be identified and addressed early from multiple perspectives and transitions into outpatient structures can be arranged where necessary. Taking the social environment of children and adolescents into consideration when examining emotional and behavioral problems is consistent with the principle of multisystemic therapy [44]. Efficacy of this digital aftercare in the intervention group is compared to an active control group with regular aftercare within a randomized controlled trial using a naturalistic parallel group study design. A longitudinal pre-post-follow-up assessment at admission, discharge, and 8, 24, and 36 weeks after discharge supplemented by a daily smartphone-based ambulatory assessment from a triadic perspective of patients, parents, and teachers allows for a close investigation of between-person differences and within-person processes in patients’ daily lives. The supplemented ambulatory assessment moreover allows ecologically valid measurements in participants’ daily lives, at the same time reducing retrospective bias [45]. Answers from the ambulatory assessment are graphically processed for the therapists on a weekly basis during aftercare [37]. The assessments are based on the comprehensive conceptual model of outcomes of mental health care for children and adolescents [46]. Accordingly, treatment success and service effectiveness are not only considered as symptom reduction on the individual level, but also related to interactions with the environment in different settings (e.g., home, school, community) [46]. The model proposes five outcome domains (symptoms, functioning, consumer perspectives, environments, systems) in which impact of interventions can be seen [46]. To address these domains, the assessments in DigiPuR include a multi-perspective view on the reintegration phase and instruments used for assessment reflect the domains. The comprehensive assessment serves to examine the general applicability and acceptance as well as the efficacy of this new digital aftercare program for the development of structures specifically for aftercare. Implementing DigiPuR in clinical practice thereby requires a balancing of scientific comparability and practical feasibility in the conception of the design as well as in the scientific evaluation, focusing on aftercare which is flexible and tailored to the needs of the individual patients.

### Hypotheses

DigiPuR examines whether the reintegration of children and adolescents after their stay in a psychiatric hospital can be facilitated by digitally supported, cross-contextual aftercare. Four hypotheses emerge for the primary outcomes. First, we hypothesize readmission rates to be lower at the post- and follow-up time points in the intervention group than in the control group (treatment-as-usual; TAU). Second, we hypothesize satisfaction with treatment to be higher at the post-time point in the intervention group than in the control group. Third, we hypothesize health-related quality of life to develop better over time (pre-post follow-up) in the intervention group than in the control group. We do not necessarily expect the intervention group to improve in absolute values over time, as measures can decline after discharge, indicated by high rates of readmissions [8, 10]. Rather, we expect the intervention group to develop better over time than the control group, meaning that even a stabilization of values might be considered a success. Fourth, we hypothesize symptom severity to develop better over time (pre-post follow-up) in the intervention group than in the control group. In line with the last two hypotheses, a better development in the intervention group than in the control group is also expected for the secondary outcomes on stress, self-efficacy, and perceived competence, including ambulatory assessment on well-being, affect, sleep quality, school situation, and social relationships.

## Methods

This manuscript includes information on all Standard Protocol Items: Recommendations for Interventional Trials (SPIRIT, checklist in Additional file 1) [47].

### Participants and recruitment

**Fig. 1:**
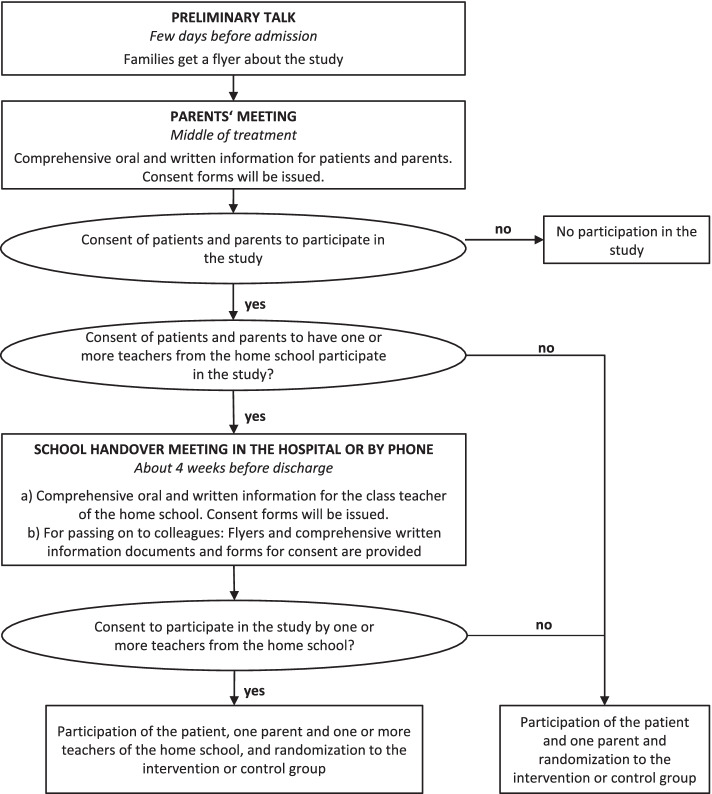
Recruitment process

Recruitment of participants takes place at the Department of Child and Adolescent Psychiatry, Psychosomatics and Psychotherapy at the University Hospital of Psychiatry and Psychotherapy Tuebingen, Germany, and is embedded in the regular routines of the hospital (see Fig. 1). Potentially, all children and adolescents who are patients in the department’s child therapy ward, adolescent therapy ward, day hospital for children, or day hospital for adolescents; a relevant primary attachment figure (a parent or caregiver from a residential group); and optionally teachers from the child’s or adolescent’s school are eligible to participate in the study. The children and adolescents are usually between 6 and 18 years old. We aim to investigate *N* = 50 triplets (*n* = 25 children/adolescents, *n* = 25 parents, and *n* = 25 teachers in each group). While sufficient German language skills are an inclusion criterion, the specific indication for partial or full inpatient treatment, type of diagnosis, or time of discharge are not. Transition to regular outpatient treatment is also not an exclusion criterion, as DigiPuR aims to form a good transition to outpatient structures in coordination with all involved parties. The study exclusion criteria are kept minimal to allow as many patients as possible to access the aftercare program. DigiPuR study staff are in weekly communication with the therapists in the department’s therapy wards and day hospitals to identify participants. In case of acute psychological strains during the study, the procedure is analogous to conventional psychotherapy with emergency presentation at the responsible hospital. If emergency care result in an inpatient stay of less than 2 weeks, participation in the study is continued (ambulatory assessment continues, intervention components resume after discharge) and, in case of more than 2 weeks, discontinued. A pilot phase of the study was conducted in early 2021, followed by recruitment from March 2021 to June 2022.

### Randomization and blinding

Participants are assigned to the intervention or control group after having given written informed consent to participate in the study. Children or adolescents, their parents, and their teachers are always assigned to the same group. A randomization list with equal proportions of both groups was generated with the software R (based on the function “sample”) [48] before the start of the study. Participants are successively assigned to one of the two groups according to the order of their recruitment (date of written informed consent) using the randomization list. Only after inclusion in the study, DigiPuR study staff look up the assignment in the randomization list and record the assignment in the coding list, which is kept locked in a cabinet and only accessible to the study management. They inform therapists about the group assignment by e-mail and participants during the technical briefing by a video or phone call or on site at the hospital. Blinding of participants, case-leading therapists, and conducting study staff is not possible due to the different nature of treatment in the two groups. Data evaluation, however, is blinded, as group allocation is coded without knowledge of the data analysts.

### Interventions

**Fig. 2:**
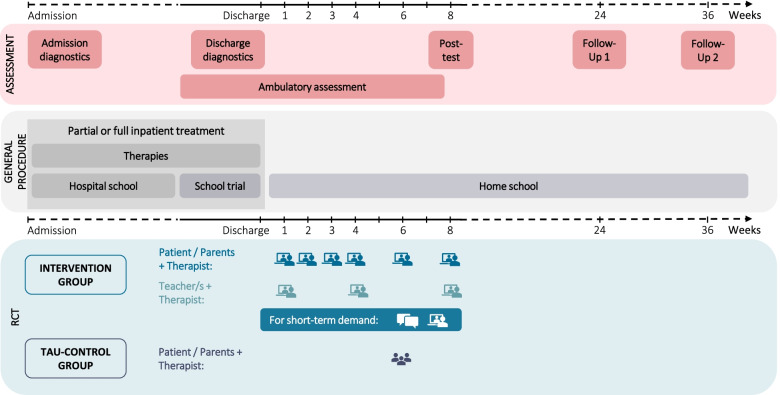
Time schedule of the study for both groups.  = video/phone call.  = messenger  = aftercare appointment at the hospital

In a naturalistic parallel group study design, DigiPuR compares a new digitally supported aftercare in the intervention group with TAU aftercare in the control group (see Fig. 2). Aftercare is provided in both groups, with aftercare in the control group being the same as the procedure for non-participation (i.e., TAU). Case-leading therapists working in the partial or full inpatient sector conduct the aftercare in both groups but are otherwise not involved in planning and evaluating the study. Documentation of aftercare appointments in both groups is handled digitally by the case-leading therapists using predefined input fields on relevant aspects in the hospital’s internal software programs. For both aftercare treatments following the partial or full inpatient stay, no additional risks are to be expected. There is no anticipated harm and no compensation for participating in the study. If there are any queries or complaints about the study or the data management, participants can contact the appropriate study-independent office by e-mail or telephone at any time using the contact details on the participant information form. DigiPuR study staff work in the research department and are at no time involved in the therapeutic process, but are contact persons for questions regarding, for example, technical difficulties of participants or the case-leading therapists.

#### Intervention group

In the intervention group, children and adolescents take part in six video calls with the case-leading therapist of the previous partial or full inpatient hospital treatment. These video calls take place weekly during the first 4 weeks after discharge, then every 2 weeks until 8 weeks after discharge and last up to 50 min, depending on the individual needs of the patient as determined by the therapists. Participants take part in these six video calls from their homes. The case-leading therapist decides whether parents and, if required, responsible persons from external support systems such as youth services, outpatient psychotherapists, psychiatrists, and pediatricians also participate in the video calls. Content of the video calls depends on the case and is linked to previous partial or full inpatient behaviorally oriented psychotherapy with focus on transfer of learned strategies into daily routines. A weekly goal is worked out with the child or adolescent in each video call and written down on a worksheet so that its achievement can be assessed retrospectively at the next session. The case-leading therapist deciding on duration, participants, and content allows the aftercare program to be sufficiently flexible and well adapted to the needs of the patients. Reducing the frequency of aftercare from weekly to biweekly video calls aims to gradually strengthen the patient’s autonomy and allows support in the home environment with personal contact to the therapist, but without time-consuming and costly travel time.

The child’s or adolescent’s and parents’ agreement provided, additionally one or more teachers from the child’s or adolescent’s school participate in up to three 30- to 45-min video or telephone calls, separate from the family’s appointments, with the hospital’s case-leading therapist in the period up to 8 weeks after discharge. The German handbook *Psychische Erkrankungen im schulischen Umfeld* (Mental disorders in the school environment) is intended to support teachers additionally by providing information and support beyond the three calls. We developed this handbook primarily for schools in the state of Baden-Wuerttemberg, Germany in response to the internally identified needs of teachers for more knowledge about mental disorders and how to deal with students with mental disorders. It covers the following topics: (1) knowledge about mental disorders, (2) dealing with mental disorders in daily school life, (3) returning after a psychiatric hospitalization, (4) dealing with mental emergencies, (5) finding an appropriate attitude, (6) hints for talking to affected students, (7) taking care of yourself, and (8) school programs for health promotion (publication of the handbook is planned after the end of this study; for more information, please contact the corresponding author). The contents of the handbook are possible subjects of video or telephone calls with teachers, with different key aspects depending on the specific case.

All children or adolescents, parents, and teachers in the intervention group can send a direct message via messenger function to the case-leading therapist via the app *movisensXS* [49], individualized by the authors as the PANDA-App (Psychiatrische Aufenthalte – Nachsorge Durch App, Psychiatric Stays – Aftercare Through App). Messages can refer to, for example, content issues or organizational matters, but do not contain personal data such as names or locations due to data security restrictions. The case-leading therapists receive these messages via their professional e-mail address and can reply to messages in the browser after logging in to the Movisens platform. This messenger service must, however, not be used for emergency contacts, as those must be made via the regular emergency number of the responsible hospital.

#### Control group (TAU)

In the control group, children and adolescents and their parents are usually given a 50-min follow-up consultation with the case-leading therapist from the previous partial or full inpatient treatment in the hospital about 6 weeks after discharge. This corresponds to the usual aftercare treatment (TAU) in the Department of Child and Adolescent Psychiatry, Psychosomatics and Psychotherapy at the University Hospital of Psychiatry and Psychotherapy Tuebingen, Germany.

### Evaluation

Evaluation of DigiPuR includes a pre-post-follow-up assessment and an ambulatory assessment phase (see Fig. 2). All assessments are carried out in the same manner for the intervention and the control group. Adherence of participants regarding the assessment data is continuously and timely monitored by checking the return of questionnaires and the documentation of aftercare appointments. In case of multiple missing data, study staff inquire by phone. There are weekly project meetings to discuss trial conduct and progress. Internal reports document and provide information on project progress on a quarterly basis. As the trial is a low-risk intervention, there is no trial auditing.

#### Pre-post-follow-up assessment

Admission and discharge assessment as part of the standard hospital assessment are carried out on paper questionnaires (in exceptions where necessary online) through trained staff during the partial or full inpatient hospital stay in the period from preliminary talk to admission until 1 week after admission and in the period from 1 week before discharge until discharge (pre). For the post, follow-up 1, and follow-up 2 assessment (8, 24, and 36 weeks after discharge), the participants receive an e-mail containing a link for an online survey on the SoSci Survey platform [50], which they can respond to at home via the browser of their computer, tablet, or smartphone within 1 week. At each time point of assessment, the survey lasts no longer than about 45 min.

#### Ambulatory assessment

**Fig. 3:**
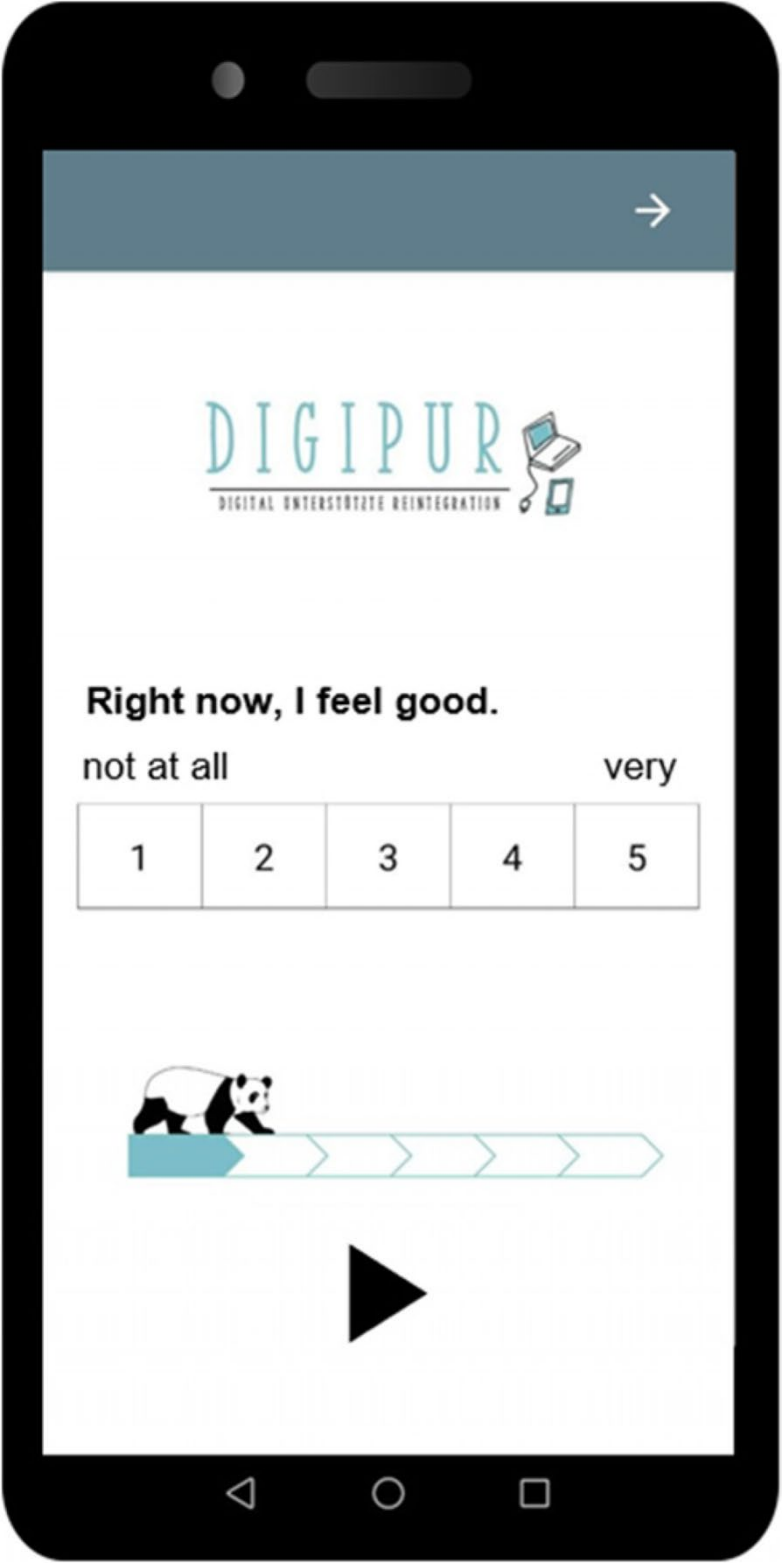
Display of the app with one example item from the ambulatory assessment and with the identification figure PANDA

**Fig. 4:**
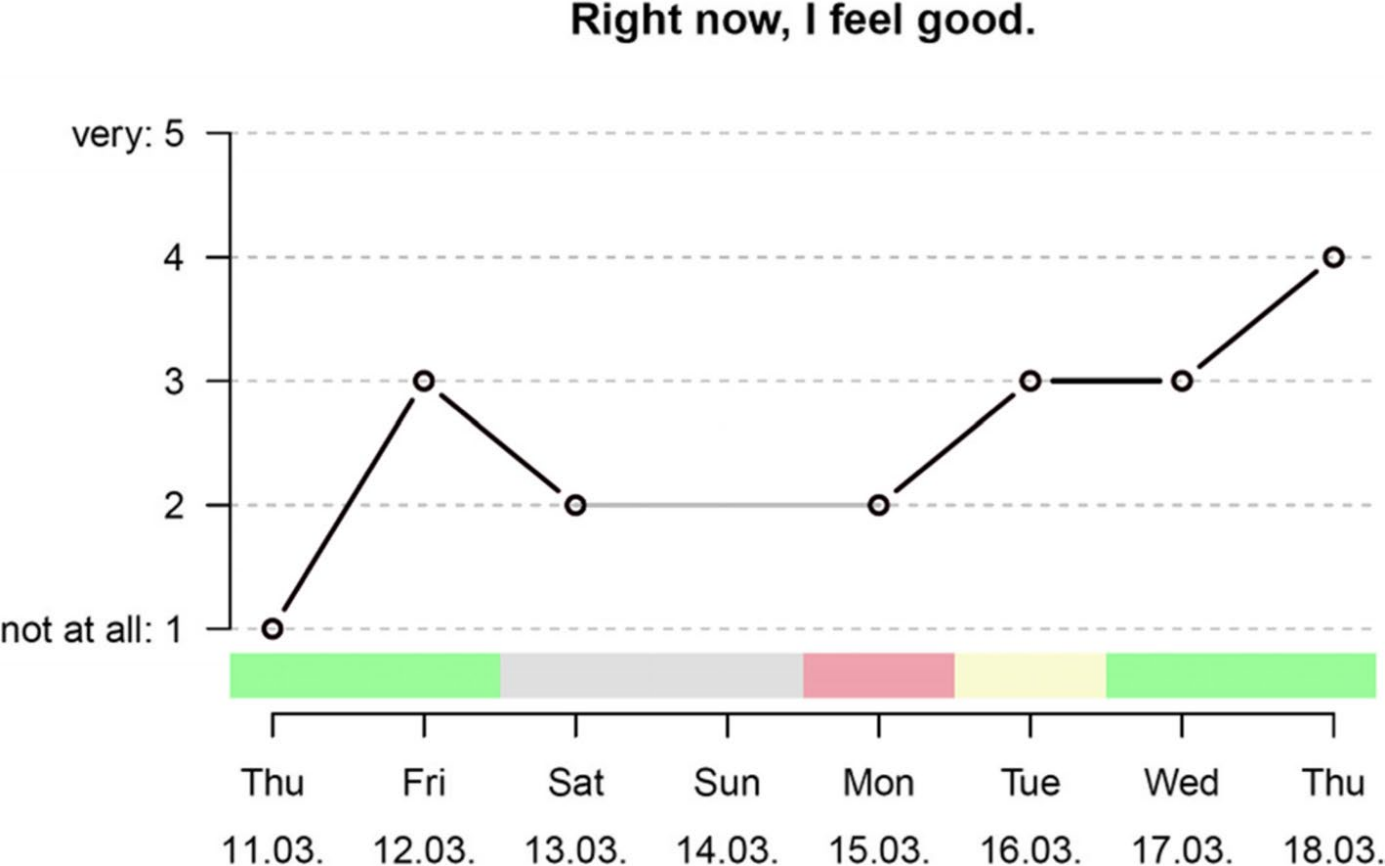
Sample excerpt from the weekly graphical evaluation of the ambulatory assessment data for the therapist. o data point.  missing. Color coding for school attendance (green = yes, yellow = partial, red = no, gray = no school day)

The ambulatory assessment consists of daily questions about well-being, affect, sleep quality, the school situation, and social relationships that children and adolescents (30 items), the participating parent or caregiver (17 items), and, if teachers participate, a teacher (10 items), answer on their personal or a study smartphone via the app *movisensXS* [49] (see Fig. 3). The children’s and adolescents’ items are presented in written and audiovisual form, so that children who are not yet able to read confidently can have the items read aloud. Ambulatory assessment takes place every day between 5 and 9 o’clock in the evening from 2 weeks before discharge to 8 weeks after discharge and takes a maximum of 5 min per day. Teachers answer the items only on school days and have a larger time frame to respond to increase adherence by being more flexible. Children and adolescents, parents, and teachers are reminded of the daily survey by alarms and can postpone alarms three times. The data collected in the ambulatory assessment is evaluated individually on a weekly basis by summarizing all answers and preparing them graphically (see Fig. 4). The case-leading therapist can use this overview of the participants answers during the last week(s) as a basis for the next regular video call.

With the start of the ambulatory assessment, all participants receive a short technical briefing, in which the app *movisensXS* [49] is installed on the participant’s Android smartphone or a study smartphone. When using their own smartphones, the participants can be reimbursed for data used for the daily surveys. After installation of the app *movisensXS* [49], all technical components of the study are briefly explained and tested: (1) the app *movisensXS* [49] with messenger function, (2) the video phone program VidyoConnect [51], (3) the university hospital’s own cloud service for the pseudonymized worksheets of the weekly goals and other therapy materials, and (4) the online platform SoSci Survey [50] for post- and follow-up surveys. Despite the intuitive and simple handling of all components, all participants receive written, illustrated instructions. For further questions or in case of technical difficulties, the study contact details (e-mail address and telephone number) are also included in the instruction.

#### Instruments

The instruments used in DigiPuR (see Table 1) are chosen to cover the five domains (symptoms, functioning, consumer perspectives, environments, systems) of the comprehensive conceptual model of outcomes of mental health care for children and adolescents [46]. The instruments and scales used in the pre-post-follow-up assessment and the ambulatory assessment are briefly described in the following, sorted according to the five domains. Item formulations were adapted where necessary so that they are understandable and suitable for both children and adolescents. Items are only presented if their content is relevant to the respective group (e.g. items on satisfaction with the program for video calls only when it was used). Primary outcomes are number of readmissions, satisfaction with treatment, health-related quality of life, and symptom severity; all others are secondary outcomes (see Table 1). As there is a great heterogeneity of diagnoses in the patient sample, the evaluation aims to assess the reintegration phase and the well-being of patients globally and across disorders. General constructs relevant to different disorders were selected and instruments were used for comparability across the age range.

**Table 1:** Overview of assessed outcomes, assigned domains, instruments, and raters at the different study time points

Outcome	Domain [46]	Instrument or scale	Rater	Study period				
				Enrollment	Allocation	Post-allocation		
				Admission	Ambulatory assessment (2 weeks before discharge until 8 weeks after)	Pre (discharge)	Post (8 weeks after discharge)	Follow-up (24 and 36 weeks after discharge)
Symptom severity	Symptoms	DISYPS-III SCREEN [52]	CA, P*, T*	x		x	x	x
Stress vulnerability and coping strategies	Symptoms (part 1), functioning (part 2)	Adapted from SSKJ-3-8R (parts 1 and 2) [53]	CA	x		x	x	x
Self-efficacy	Functioning	WIRKSCHUL [54], WIRKSOZ [54], WIRKLEHR [54], WIRKELTERN (self-development)	CA, P, T	x		x	x	x
Health-related quality of life	Consumer perspectives, environment	KIDSCREEN-27 [55]	CA, P*	x		x	x	x
Occupational well-being	Environment	Emotional exhaustion [56], enthusiasm for teaching [56]	T			x	x	x
Satisfaction with treatment	Consumer perspectives	Adapted from FBB [57]	CA, P, T, TH				x	
Satisfaction with technical components	Consumer perspectives	SUS [58], self-development	CA, P, T, TH				x	
Expectation of change	Consumer perspectives	Adapted from patient’s expectation of change [59]	CA, P			x		
Parental stress	Consumer perspectives, environment	EBI [60]	P	x		x	x	x
Parental strain	Consumer perspectives, environment	BSCL [61]	P	x				
Competence self-concept in dealing with students	Environment	Competence self-concept in dealing with students [62]	T			x	x	x
Professional competence in dealing with students with mental disorders	Environment	Self-development, Selection of different items from [54, 56, 63–66]	T			x	x	x
Number readmissions, and general information	All domains	Hospital documentation, self-development	P, T, TH	x		x	x	x
General well-being	Symptoms	Self-development	CA, P, T, P*, T*		x			
Affect	Symptoms	Affect [67]	CA, P		x			
Sleep quality	Symptoms	Sleep quality [67]	CA		x			
School absenteeism	Functioning	Self-development	CA		x			
School day	Functioning	Academic success [68]; adapted from KIDSCREEN-27 [55]	CA, T*		x			
Relationship with peers, parents and teachers	Consumer perspectives, environment	Peer relatedness [69], adapted from KIDSCREEN-27 [55]	CA, P*, T*		x			
Support from the hospital	Systems	Self-development	T		x			

*Note*. All items presented in German. *CA* children and adolescents, *P* parent, *T* teacher, *TH* therapist. *Assessed regarding the child or adolescent

##### DISYPS-III SCREEN (Diagnostic System for Mental Disorders according to ICD-10 and DSM-5 for Children and Adolescents - III Screening questionnaire)

The DISYPS-III SCREEN [52] contains five superordinate scales for overall symptomatology, internalizing and externalizing behaviors, contact behavior, and functional impairment and distress, as well as seven disorder-specific subscales. In the present study, overall symptomatology is utilized to evaluate the symptom severity from the perspective of the patients (self-evaluation questionnaire SBB; 55 items), parents, and teachers (external evaluation questionnaire FBB; 55 items). In addition to the superordinate scales, the corresponding disorder-specific subscales are analyzed. The manual reports satisfying psychometric properties (internal consistency of the overall symptomatology: Cronbach’s *α* = .91 for SBB and *α* = .92 for FBB, disorder-specific subscales between *α* = .49 and *α* = .85).

##### Questionnaire based on the SSKJ 3-8R (Questionnaire for the Survey of Stress and Stress Coping in Childhood and Adolescence – Revision)

The SSKJ 3-8R [53] contains three parts: (1) stress vulnerability, (2) stress coping strategies, and (3) stress symptomatology and well-being. In the present study, a self-developed questionnaire based on items of the SSKJ 3-8R measures children and adolescents’ (1) stress vulnerability with seven items on seven different situations and (2) stress coping strategies with 30 items on five scales. Deviating from the original version, the items on stress coping strategies are answered only in relation to one situation, which the children and adolescents choose beforehand from the previous stressful seven situations. The manual reports for the original version satisfying psychometric properties (internal consistencies between *α* = .67 and *α* = .89; retest reliabilities between *r*_*tt*_ = .61 and *r*_*tt*_ = .82).

##### Self-efficacy scales

Self-efficacy in dealing with social demands in children and adolescents is assessed via the scale WIRKSOZ [54] with eight items (internal consistency *α* = .60). School-related self-efficacy of children and adolescents is assessed via the scale WIRKSCHUL [54] with seven items (internal consistency ranges from *α* = .70 to *α* = .73). For the assessment of teachers’ self-efficacy in relation to students with mental disorders and in relation to the student to be reintegrated, 5 of the total 10 items of the Teacher Self-Efficacy scale, WIRKLEHR [54], were selected for the present study and adapted thematically in each case. Internal consistency of the original WIRKLEHR scale ranges from *α* = .76 to *α* = .81. To assess parents’ social self-efficacy in dealing with their child, the first 8 of the total of 10 items from the WIRKLEHR scale were thematically adapted and combined to form the WIRKELTERN scale.

##### KIDSCREEN-27

The KIDSCREEN-27 [55] measures the health-related quality of life of children and adolescents with 27 items on five dimensions (physical well-being, psychological well-being, relationship with parents and autonomy, peers and social support, school environment). In the present study, health-related quality of life is assessed from the perspective of both the child or adolescent and the participating parent. The manual reports satisfying psychometric properties (internal consistency between *α* = .80 and *α* = .84; intraclass correlation coefficients for retest reliability between .61 and .74).

##### Emotional exhaustion and enthusiasm for teaching

The scales emotional exhaustion (4 items; internal consistency between *α* = .74 and *α* = .82) [56] and enthusiasm for teaching (3 items, number 4, 5 and 6 selected; internal consistency between *α* = .69 and *α* = .81) [56] are used in the present study to assess teachers’ occupational well-being (see also [70]).

##### Questionnaire based on the FBB (Questionnaire for the Assessment of Treatment)

The FBB [57] is a German questionnaire used for therapy evaluation and quality assurance of psychotherapeutic and psychiatric treatments of children, adolescents, and families in research and practice. In addition to overall satisfaction with the treatment, outcome quality (treatment success) and process quality (course of treatment) are measured as subjective assessments of treatment. In the present study, four self-developed versions (patients: 20 items, parents: 21 items, therapists: 27 items, teachers: 14 items) adapted from items of the FBB are used, in which the word “treatment” was specified by “aftercare treatment”. In the patient version, item number 13 on video recordings was deleted because there are no video recordings in the present study. Item number 5 was added to the therapist version, asking about relationships in school in addition to relationships in the family. In addition, a version for teachers was designed from 14 items of the parent version of the FBB. The manual reports for the original versions satisfying psychometric properties (internal consistency between *α* = .63 and *α* = .94 for all raters; retest reliabilities for patients and parents ranged from *r*_*tt*_ = .36 to *r*_*tt*_ = .79).

##### SUS (System Usability Scale)

Satisfaction with the technical components is assessed among children and adolescents (23 items), parents (23 items), teachers (22 items), and therapists (15 items; no items regarding the app). The 10 items of the widely used technology-independent questionnaire SUS [58] were thematically adapted to the aftercare app and the program for the video calls, both used in the present study (e.g., “I thought the aftercare app was easy to use”). A study of the factor structure of the SUS reported an internal consistency of *α* = .92 [71]. Further self-developed items asked about things that did not work (all raters), preferences in aftercare (children and adolescents, parents, therapists), and the usefulness and inclusion of the weekly graphic evaluation (therapists).

##### Expectation of change

Patients’ and parents’ expectation of change regarding the aftercare is assessed as a covariate with 4 thematically adapted items (e.g., “I think that through DigiPuR I will cope better with my daily life”) from the scale *Patient’s expectation of change* of the questionnaire for measuring common factors in psychotherapy (FERT; factorial reliability of the original scale with 8 items ρϲ = .94) [59].

##### EBI (Eltern-Belastungs-Inventar)

The EBI [60] is the German version of the Parenting Stress Index [72] and measures parental stress with 48 items. In the present study, parental stress is assessed by means of the total scale and the twelve subscales. The manual reports satisfying psychometric properties (internal consistency of the total score *α* = .95 and of the subscales between *α* = .61 and *α* = .83; retest reliability *r*_*tt*_ = .87 for total scale).

##### BSCL (Brief Symptom Checklist)

The BSCL [61] contains 53 items to assess psychological strain in self-judgment on three global indices and nine scales. In the present study, the BSCL is used as a covariate to assess psychological strain in parents. The manual reports satisfying psychometric properties (internal consistency for the total score *α* = .97 and for all scales *α* ≥.72; retest reliability *r*_*tt*_ = .87 for the total score and *r*_*tt*_ ≥.64 for all scales).

##### Competence self-concept in dealing with students

Teachers’ competence self-concept in dealing with students is assessed with four items [62]. A recent study reported an internal consistency of *α* = .77 [73].

##### Professional competence in dealing with students with mental disorders

The professional competence of teachers in dealing with students with mental disorders is exploratively examined. Orientated on the framework model of teachers’ professional competence [74], seven competence facets relevant to the present study (professional, pedagogical-psychological, organizational, and counseling knowledge as well as motivational orientations, self-regulation, and beliefs/values attitudes) were thematically adapted: knowledge about mental disorders (5 items), knowledge about dealing with the student with mental disorders (11 items), knowledge about mental emergencies (3 items), knowledge about counseling of affected persons (8 items), motivation and self-efficacy (13 items), self-regulation (8 items), and responsibility and relationship with students with mental disorders (13 items). For this purpose, items of different studies from the field of Mental Health Literacy [63–65] and from teacher survey studies [54, 56, 66] were selected, thematically adapted where necessary as well as own items were developed.

##### Hospital documentation on general information

General and professional information about participants is obtained from hospital documentation and anamnesis forms for families (e.g., diagnoses, medications, level of psychosocial functioning, duration of treatment) and is collected from teachers at study inclusion (e.g., years in the profession, type of school, teaching load, professional contact with students with mental disorders). Further information for a health economic evaluation is obtained by analyzing controlling data (e.g., percentage of readmissions; travel times; contact via messenger) and the therapist’s documentation (e.g., worksheets on weekly goals; missed appointments; cooperation with support systems; implementation of therapy suggestions).

##### Ambulatory assessment

Ambulatory assessment items contain information on children and adolescents’ general well-being (1 item; self-development), affect (12 items) [67], sleep quality (2 items) [67], school absenteeism (2 items; self-development), school day (4 items; three items to perceived success at school [68] and one item adapted from the KIDSCREEN-27 [55]), and relationship with peers (4 items) [69], parents (4 items; adapted from [69]), and teachers (1 item; adapted from the KIDSCREEN-27 [55]). Parents make corresponding statements in the ambulatory assessment about their own general well-being (1 item; self-development) and affect (12 items) [67], as well as about their child’s general well-being (1 item; self-development) and their relationship with the child (3 items; adapted from [69]). Teachers make statements in the ambulatory assessment about the support they receive from the hospital (1 item; self-development) and about contact with the student being reintegrated (filter question). If there was contact with the student, teachers make statements about their general well-being, their well-being in their role as a teacher, and the well-being of the student (1 item each; self-development) as well as about the teacher-student relationship (1 item; adapted from the KIDSCREEN-27 [55]) and the school day (4 items; three items adapted from [68] to perceived success of the child at school and one item adapted from the KIDSCREEN-27 [55]). Additional comments or observations about the day can be indicated by parents and teachers in an open response format at the end of each assessment.

### Statistics

#### Calculation of the sample size

It is planned to include a total of *N* = 50 triplets (total sample approximately 150 persons) with 50 children and adolescents (*n* = 25 per group), one attachment figure (parent or caregiver) each and, if possible, at least one associated teacher each. Estimation of sample size is based on recently published randomized controlled trials, in which the efficacy of digital treatment for children and adolescents with mental disorders is compared with control conditions [75–77] and on the anticipated number of discharges of the department during the course of the study (average treatment duration of 3 months with approximately 10 children per ward).

#### Statistical analyses

All analyses are conducted using R software [48]. All outcome variables measured pre-post-follow-up will be analyzed with separate mixed ANOVAs. Preprocessing of the data and calculation of individual values will be done according to the respective manual. Predictors will be group (intervention vs. control) and time point, as well as their interaction. Treatment success would be demonstrated by a significant interaction effect of group and time point, being shown, through adequate post hoc test, to be due to different time-effects in the two groups. We do not necessarily expect the intervention group to improve in absolute values over time, as measures can decline after discharge. Rather, we expect the intervention group to develop better over time than the control group, meaning that even a stabilization of values might be considered a success. This better development in the intervention group than in the control group should be evident in the information provided by patients themselves, as well as by parents and teachers.

The successful reintegration into daily (school) life is conceptualized besides the pre-post-follow-up assessment by the data collected in the ambulatory assessment. All ambulatory assessment data will be analyzed with multi-level models [78]. We expect the reports from patients, parents, and teachers in the intervention group to show a significantly more positive trend over time in terms of their general well-being, affect, sleep quality, school absenteeism, situation in class, and the relationship with parents, peers, and teachers, than those in the control group. Statistically, these effects are shown by a significant interaction of the within-person time trends of these reports with group membership, being due to participants in the intervention group developing better (or less bad) than participants in the control group. Variables will be adequately centered, and appropriate fixed and random effects will be included in the models.

Few missing data are expected due to ongoing monitoring. Missing data from single items of the standardized questionnaires will be handled appropriately according to the instructions in the manual. In case of complete questionnaires missing for a participant, this participant will be excluded from all ANOVAs containing this questionnaire. As multi-level models can handle missing data quite well, if they are at least missing at random, no data will be excluded from those analyses. However, appropriate variables (e.g., age, symptom severity) will be used to investigate if the missingness structure can be explained by them, rendering it to be not at random. In that case, those variables will be included in the according models to account for this non-randomness.

Due to the confidentiality of patient health data, data sets cannot be made publicly available. Access is only granted to study management who supervise data management and have a duty of confidentiality.

#### Data security and storage

Procedure for data management was approved by the data protection officer and the IT security officer of the University Hospital Tuebingen, Germany. The collection and storage of all data is pseudonymized and stored for 10 years. All questionnaires are labeled with an anonymous personal code of the participant. Data can only be assigned to individuals based on a coding list, which is continually updated by hand, kept locked, and only accessible to study management. The coding list will be destroyed at the end of the study.

##### Pre-assessment

All information used in paper form for the study at admission and discharge will be kept in the participating child’s or adolescent’s medical record and is additionally entered pseudonymously into a spreadsheet file, so that the information can only be assigned to the other data by the personal code.

##### Post- and follow-up assessment

Data collected with SoSci Survey [50] at post and follow-up is protected by SSL encryption (HTTPS) Extended Validation (EV) both when filling in the questionnaire and when retrieving the collected data. Data is hosted on an ISO27001-certified server in Germany. The provider protects against data loss and access by third parties by means of a firewall.

##### Ambulatory assessment

Data from the ambulatory assessment will be encrypted, ensuring that the collected data cannot be accessed by unauthorized persons even if the device is lost. All data connections (i.e., both the communication between the smartphone and the server and the communication between the server and the researcher’s browser) are encrypted using 256-bit SSL encryption. Furthermore, participants are instructed not to indicate personal data that could be used to identify the children and adolescents, parents, teachers, or other persons (e.g., names, locations, etc.), neither when answering the questions nor in the messages in the app *movisensXS* [49].

##### Video calls

The program VidyoConnect [51] for the video calls in the intervention group is a standard software certified by the university hospital. No data is recorded during use. Participants are instructed not to produce and distribute any audio or visual material of the sessions.

##### Cloud

The firewall-protected cloud of the university hospital is used for the exchange of files with participants. It meets particularly high security requirements and is approved for the storage of anonymized or pseudonymized patient data. Participants receive a link with a password to their own folder in which the therapist can store worksheets (e.g., documentation of weekly goals) and information in PDF format. There is never a name or other identifiable information on the materials. Access to the folder in the cloud is restricted to the therapist and the respective participant.

##### Storage locations and dissemination

At the end of the study, all participant data is deleted from the servers used by *movisensXS* [49] and SoSci Survey [50] as well as the cloud of the university hospital. From this point on, the pseudonymized data of the participants are stored in the form of a spreadsheet file exclusively on the drive of the university hospital, to which only authorized staff have access, and evaluated with the help of statistical analysis software. Paper-pencil data are entered twice, compared with the help of an R-script [48] and checked for errors. Evaluation data from the online surveys are summarized into a data set by means of an R-script [48] without reference to specific individuals. Participants do not receive individual feedback on their data. Instead, the anonymized results will be published in scientific journals and presented at conferences and in the hospital. The study is monocentric and has no data monitoring committee as it is a low-risk intervention.

## Discussion

DigiPuR is a longitudinal randomized controlled trial comparing regular aftercare (TAU) in the control group with a novel, digital aftercare service in the intervention group. In the intervention group, regular video calls take place with all parties involved during the reintegration phase until 8 weeks after discharge. In addition, patients, parents, and teachers have a direct communication channel to the therapist via a secure messenger system. This is intended to stabilize treatment successes after discharge and, if necessary, to accompany the transition to outpatient services. This paper describes the naturalistic parallel group study design of DigiPuR and its evaluation. Besides pre-post-follow-up surveys, an ambulatory assessment provides ecologically valid daily data on the reintegration phase from the triadic perspective of patients, parents, and teachers. Psychological strains and exacerbations should thus be recognized at an early stage, addressed and hence readmissions reduced.

Limiting factors that should be considered in the interpretation of the results include that questionnaires in the evaluation are used across the age range of both children and adolescents. This was necessary to ensure sufficient comparability but implied adapting item formulations to a wide age range with possible limitations regarding psychometric properties of questionnaires used. Second, due to the different nature of treatment in the two groups, blinding of participants, therapists, and study staff is not possible. Data analysis, however, is blinded, as group allocation is coded without knowledge of the data analysts. Third, the time required for aftercare appointments and the assessments may be a barrier to study participation and is likely to be weighed against the personal benefits of increased support during the reintegration phase. However, the decision for study enrollment takes place before randomized group assignment, so any potential bias should not affect group comparisons.

Results will show whether DigiPuR can increase participants’ health-related quality of life and reduce a worsening of symptoms as well as rehospitalizations. It is expected that the digitally supported, seamless, and regular aftercare appointments involving family and school attachment figures in the intervention group will contribute to a successful reintegration. DigiPuR is designed to improve cross-sectoral child and adolescent psychiatric health care, but in doing so, also to provide further training in this area for teachers in the educational system. Expanding the spectrum of traditional services by establishing digital media as contemporary, widespread, and practicable tools in daily clinical practice can improve child and adolescent psychiatric care because a wider spectrum offers more treatment options in general, as well as more possibilities to adapt treatment to the characteristics and needs of individual patients [34, 79]. The COVID-19 pandemic, as an unforeseen crisis that came along with considerable mental stress for many children and adolescents [80–83], may simultaneously act as an accelerator in establishing E-Mental-Health in health care [84]. In this regard, the expansion of digital infrastructure that occurred in the wake of the COVID-19 pandemic and the associated broader use of digital media can be expected to have a beneficial impact on DigiPuR. Barriers for using digital media can be expected to decrease further, willingness to communicate without being tied to a specific location will increase, and technical skills will improve. E-Mental-Health is expected to advance mental health care by being accessible, available, attractive, and cost-effective [85] and should go along with collaborative research from science and practice in naturalistic settings [86]. In case of efficacy and successful implementation of DigiPuR in clinical daily processes, an integration of DigiPuR into regular care is possible and planned.

### Trial status

The trial is ongoing (10-29-2021, version 1, start of recruitment: March 2021, estimated end of recruitment: June 2022)

## Supplementary Information

**Additional file 1.** SPIRIT checklist.

## Data Availability

Data cannot be made available due to confidentiality of patient health data. However, the statistical code and all study materials will be available from the corresponding author on reasonable request, and any questions about the procedure are welcome. Contact for more information on the study: digipur@med.uni-tuebingen.de or 004970712962535.

## Abbreviations

BSCL: Brief Symptom Checklist
DigiPuR: Study name (Digital gestützte Psychotherapie und Reintegration – Digitally supported psychotherapy and reintegration)
DISYPS-III SCREEN: Diagnostic System for Mental Disorders according to ICD-10 and DSM-5 for Children and Adolescents - III Screening questionnaire
EBI: Eltern-Belastungs-Inventar (German version of the Parenting Stress Index)
FBB: Fragebogen zur Beurteilung der Behandlung (German Questionnaire for the assessment of treatment)
KIDSCREEN-27: KIDSCREEN questionnaire with 27 items
PANDA: App name in the ambulatory assessment
SSKJ 3-8R: Fragebogen zur Erhebung von Stress und Stressbewältigung im Kindes- und Jugendalter – Revision (German Questionnaire for the Survey of Stress and Stress Coping in Childhood and Adolescence)
SUS: System Usability Scale
TAU: Treatment-as-usual
